# Mitochondrial Regulation of Macrophage Response Against Pathogens

**DOI:** 10.3389/fimmu.2020.622602

**Published:** 2021-02-17

**Authors:** Subhadip Choudhuri, Imran Hussain Chowdhury, Nisha Jain Garg

**Affiliations:** ^1^ Department of Microbiology and Immunology, University of Texas Medical Branch (UTMB), Galveston, TX, United States; ^2^ Institute for Human Infections and Immunity, UTMB, Galveston, TX, United States

**Keywords:** mitochondria, metabolism, macrophage, innate immunity, cGAS-STING, mitophagy, noncoding RNA

## Abstract

Innate immune cells play the first line of defense against pathogens. Phagocytosis or invasion by pathogens can affect mitochondrial metabolism in macrophages by diverse mechanisms and shape the macrophage response (proinflammatory *vs*. immunomodulatory) against pathogens. Besides β-nicotinamide adenine dinucleotide 2'-phosphate, reduced (NADPH) oxidase, mitochondrial electron transport chain complexes release superoxide for direct killing of the pathogen. Mitochondria that are injured are removed by mitophagy, and this process can be critical for regulating macrophage activation. For example, impaired mitophagy can result in cytosolic leakage of mitochondrial DNA (mtDNA) that can lead to activation of cGAS–STING signaling pathway of macrophage proinflammatory response. In this review, we will discuss how metabolism, mtDNA, mitophagy, and cGAS–STING pathway shape the macrophage response to infectious agents.

## Introduction

Macrophages (M*φ*) are innate immune cells that reside in almost all of the tissues in the body. Two major lineages of M*φ* include the blood-circulating monocytes originating from the myeloid progenitor cells in the bone marrow (BM) and those derived from the yolk sac (1). M*φ* of both lineages are capable of responding to microbial invasion and other stimuli through production of proinflammatory molecules *e.g.* tumor necrosis factor (TNF)-α, interleukin (IL)-6, nitric oxide (NO), and reactive oxygen species (ROS), recruitment of immune cells, and antigen presentation to T cells for the initiation of adaptive immunity (2). M*φ* may also play a role in resolution of inflammation and tissue injury by upregulating the anti-inflammatory mediators and scavenging receptors (*e.g.* mannose receptor), phagocytizing cellular debris, and secreting molecules [*e.g.*, metalloproteinase (MMP)-2, MMP-9, tumor growth factor (TGF)-*β*] to induce collagen production and scar formation associated with tissue repair and wound healing (3, 4). In recent studies, mitochondrial health and metabolic status have been recognized as drivers of M*φ* response (5). For example, a switch in energy production from mitochondrial oxidative metabolism towards glycolysis elicits M*φ* proinflammatory polarization (6). Bacterial lipopolysaccharide (LPS) elicits a large and transient increase in nicotinamide adenine dinucleotide (NAD^+^) levels, which supports TNF-α production; and NAD^+^ deficiency had an inhibitory effect on TNF-α release (7). Depletion of cytoplasmic NAD^+^ induces an impairment in adhesion and phagocytosis of zymosan particles (8).

A compromise in mitochondrial health in the presence of exogenous or endogenous stimuli may signal proinflammatory M*φ* response through multiple routes, including release of mitochondrial ROS (mtROS) and mtDNA that interact with multiple receptors [*e.g.* cyclic GMP-AMP synthase (cGAS)/stimulator of interferon genes (STING)] and transcription factors [*e.g.* nuclear factor kappa B (NF-*κ*B)] (7). In this context, M*φ* response may also be regulated by mitophagy involved in clearance of damaged and/or dysfunctional mitochondria (8). In this review, we will discuss how mitochondrial health *vs* mitochondrial dysfunction and compromised mitophagy shape the M*φ* response. We will also discuss the signaling pathways (*i.e.*, cGAS/STING) that link mitochondrial control of M*φ* response in health and disease.

## Diverse Functions of Macrophages

Macrophages exhibit remarkable flexibility in responding to pathogenic, environmental, and endogenous stimuli. M*φ* utilize surface and cytosolic pattern recognition receptors (PRRs) to recognize pathogen associated molecular patterns (PAMPs) that are the molecules expressed by pathogens and the damage associated molecular patterns (DAMPs) which serve as immune activators when they are exposed under conditions of stress or injury. Major classes of PRR include C-type lectin receptors, NOD-like receptors, RIG-I-like receptors, and toll-like receptors (TLRs). PRR recognition of PAMPs and DAMPs initiates complex signaling cascade and interplay of cellular mediators and transcription factors that shape the M*φ* defense against invading or intracellular pathogen or M*φ* response for removal of cellular debris left after exposure to pathogenic and injurious stimuli. In-depth discussion of PRRs’ function can be found in several recent articles (*e.g.* 9, 10).

### Proinflammatory Response

Lipopolysaccarides (LPSs), components of the outer membrane of gram-negative bacteria, are perhaps the most studied PAMPs that are recognized by TLR4 and shown to elicit proinflammatory response in M*φ*. TLRs, through a series of phosphorylation steps, signal the recruitment and degradation of proteins and kinases including TNF receptor associated factor 6 (TRAF6), myeloid differentiation primary response (MYD)88, and TGF-*β* activated kinase (TAK) that result in the induction of NF-*κ*B transcription factor for the expression of proinflammatory molecules (11). Along with the PRRs, TNF-α and type II interferon (IFN)-*γ* are the most studied cytokines shown to enhance the proinflammatory activation of the M*φ via* c-Jun N-terminal kinase (JNK)/signal transducers and activators of transcription (STAT)-1 signaling of NF-*κ*B and hypoxia inducible factor (HIF)-1*α* transcription factors (12, 13). The classical model of M*φ* activation by LPS/IFN-*γ* triggers an extensive profile of inflammatory cytokines (*e.g.* TNF-α, IL-6, IL-1β) and chemokines (such as CCL2/MCP-1, CCL3, CCL4, CCL5, CCL11, and CCL13) and are referred as proinflammatory or M1 phenotype (14). A feedback control of M1 M*φ* activation is provided by a suppressor of cytokine signaling (SOCS) inhibition of STAT-3. The proinflammatory M*φ* are also expected to generate high levels of reactive oxygen and nitrogen species which contribute to direct killing of the pathogen.

In M*φ*, NADPH oxidase is considered to be the primary source of superoxide (O_2_•^−^) that then is dismutated to peroxides, hydroxyl radical, and other forms of ROS (15). Recent studies have also addressed the role of mitochondrial respiration in contributing to ROS production in Mφ (16). Inducible nitric oxide synthase (iNOS) serves as the major source of nitric oxide (NO) utilizing oxygen and L-arginine in the process (17). The O_2_•^−^ and NO radicals, together, produce peroxynitrite (ONOO) involved in microbicidal activity. Costimulatory molecules such as cluster of differentiation (CD)80, CD86, CD64, CD16, CD32, and iNOS are used as markers of M1 M*φ*. Type I and type III interferons mediated antiviral responses that are central to host defense against viral infections are discussed in recent reviewes (18, 19).

### Immunomodulatory Response

On the other end of the spectrum is the M*φ* activation to M2 state that involves heterogenous functional profile depending upon the stimulating factors (20). For example, IL-4 produced by M*φ* and T helper type 2 (Th2) cells and IL-13 produced by various T cell subsets and natural killer (NK) cells stimulate M*φ* polarization towards immunomodulatory M2a state associated with anti-inflammatory role with production of IL-10, IL-1 receptor II (IL-1RII), and anti-helminth Th2 inflammation (21, 22). M2b regulatory M*φ* are capable of possessing both protective and pathogenic roles and are activated in response to TLRs, IL-1R or immune complexes (23). The M2c M*φ* respond to glucocorticoids, TGF-*β*, IL-10 *etc.*, and are involved in immune suppression, matrix deposition, tissue repair and tissue remodeling (24). The general mechanism underlying the M2 polarization includes the engagement of IL-4R, IL-10R, or IL-13R that signal through Janus kinase (JAK) and tyrosine kinase (TYK) to mediate an increase in STAT6 activity (25). STAT6 transcriptional activation is also enhanced by peroxisome proliferator activated receptor *γ* (PPAR-*γ*) and PPAR-*γ* coactivator 1 (PGC-1) that support the expression of genes involved in fatty acid (FA) oxidation and oxidative metabolism and have inhibitory effects on inflammatory gene transcription (26, 27). IL-4 and IL-13 also enhance the expression of the mannose receptor (CD206), dectin-1, and resistin-like molecule (RELM)-*α*, which play an important role in the recognition of fungal infections and activation of Th2 immunity (27). IL-10 activates STAT1 more than STAT3 and inhibits inflammatory cytokine (*e.g.*, TNF-α and IL-1β) production (28). Polarization of M2 M*φ* towards M1 phenotype is prevented through SOCS-3 inhibition of STAT-3. Co-stimulatory molecules, including arginase 1 (supports cell proliferation), decoy receptor 3 (inhibits Fas ligand induced apoptosis), dectin 1 (a major *β* glucan receptor), CD206, and CD163 (scavenger receptor) are used as markers of M2 M*φ*. We note that this classification is not sufficient to cover the wide range of M*φ* activation profile noted in various *in vivo* models of infection and other diseases.

### Phagocytosis

Macrophages are classical phagocytes that uptake and degrade pathogens as well as cellular debris by an actin dependent and clathrin-independent mechanism. Phagocytosis in M*φ* is activated by recognition through the complement-, Fc*γ*-, or mannose-receptors [reviewed in (29)]. The fusion of phagosome with lysosome filled with hydrolytic enzymes forms a phagolysosome where the engulfed material is digested and then disposed by exocytosis or the immunodominant peptides are loaded on to MHC. MHCII molecules translocate to cell surface for presentation of the antigen to CD4^+^T cells (30). Macropinocytosis (non-specific uptake of soluble antigens) and receptor-mediated endocytosis of soluble antigens through clathrin-coated vesicles also deliver the antigenic peptides through endosome lysosome pathway for MHCII presentation (30). Some microbes that escape from phagolysosome into cell cytoplasm (*e.g. Trypanosoma cruzi*) and others that develop intracellularly (*e.g.* viruses) are degraded by proteasomes after which immunodominant peptides bind to MHCI molecules which are recognized by CD8^+^T cells (31). Thus, as a professional antigen-presenting cell (APC), M*φ* bridge the innate and adaptive immune systems.

Besides traditional phagocytosis that is mostly reserved for uptake of pathogens, M*φ* are also involved in the uptake and clearance of apoptotic bodies. For this, M*φ* respond to “eat-me” signals including nucleotides, chemokines, and lipid phosphatidylserine on the apoptotic cells by upregulation of the mannose receptors (CD163, CD206), cytokines (TGF-*β* and IL-10), arachidonic metabolites (*e.g.*, prostaglandin E2), and pro-resolving mediators that is then followed by anti-inflammatory and repair/healing responses (32). Further details on M*φ* role in clearance of apoptotic cells can be found in a recent review (33).

## Metabolic Switch and Functional Profile of Macrophages

Mitochondrial respiration and metabolism machinery, *i.e.*, tricarboxylic acid (TCA) cycle, electron transport chain (ETC), oxidative phosphorylation (OXPHOS), and fatty acid and amino acid metabolism provide the energy house status to mitochondria. Interestingly, these same metabolic pathways, along with glycolysis and pentose phosphate pathway (PPP), influence the functional polarization of M*φ* and are discussed in brief here.

### Metabolic Switch to Glycolysis in Proinflammatory Macrophages

Newsholme et al. (34) have noted a direct correlation between the increase in the rate of glycolysis and phagocytosis upon macrophage activation by inflammatory stimuli. Since then, the advent of new reagents and mass spectrometry approaches has facilitated large scale metabolome studies. Researchers have utilized these new approaches to address how metabolic profile impacts M*φ* activation and the mechanisms employed by M*φ* to switch metabolic program in response to various stimuli. Now, we know that proinflammatory M*φ* induced by LPS/IFN-*γ* have impaired activities of the TCA, ETC, and OXPHOS pathways and rely on increased glucose uptake and glycolysis for energy needs and production of ROS/NO and proinflammatory cytokines (35). LPS-activated M*φ* also utilize the glucose metabolic intermediates for triglycerides and FA synthesis and engulf free fatty acids for triglyceride accumulation (36), which are vital for their phagocytic function (37). Glycolysis in LPS-stimulated and M1-like M*φ* is supported by upregulation of glucose transporter 1 (GLUT1), hexokinase 2 (catalyzes rate limiting first step of glycolysis) (38), ubiquitous 6-phosphofructo-2-kinase (uPFK2, produces higher concentrations of fructose 2,6-bisphosphate substrate than other isoforms), and pyruvate kinase M2 (PKM2, catalyzes final step of glycolysis) (39). M*φ* switch to glycolysis is mediated by HIF-1*α*. In normal conditions, nuclear prolyl hydroxylase 1 (PHD1) produces 2S,4R-4-hydroxyproline from *α*-ketoglutarate for post-translational hydroxylation of HIF-1*α* that is then targeted for proteasomal degradation (40). Under hypoxic conditions, PHD1 inhibition allows stabilization and activation of HIF-1*α* (40). Nuclear localization of PKM2 enhances the STAT3 phosphorylation and HIF-1*α* transcriptional activity thereby facilitating increase in proinflammatory cytokines expression (41). HIF-1*α* also stimulates the expression of GLUT1, monocarboxylate transporter 4 (MCT4), and uPFK2 and induces lactate dehydrogenase (utilizes pyruvate to make lactate) and pyruvate dehydrogenase kinase (inhibits pyruvate entry into TCA cycle) that together promote glycolysis in M*φ* (discussed in 6).

The ^13^C metabolite labeling studies suggest that TCA cycle is disrupted at distinct points in M1 M*φ*. Accumulation of succinate signals the stabilization of HIF-1*α* (42) and succinate-responsive succinate receptor 1 (SCNR1) and regulates ROS and IL-1β release in classically activated M*φ* (43). Isocitrate dehydrogenase downregulation contributes to accumulation of citrate that is deemed important for the synthesis of the anti-bacterial itaconate compound (43). Further, excess citrate is transported from the mitochondria to cytosol where it is converted by citrate lyase to acetyl CoA and used for FA synthesis in proinflammatory M*φ* (44). Other intermediates of TCA cycle, *e.g.* fumarate, succinate, and *α*-ketoglutarate are suggested to regulate the M*φ* activation profile through influencing the epigenome (45).

Pentose phosphate pathway (PPP) branches off from glycolytic pathway with formation of 6-phosphate gluconate and NADPH from glucose-6-phosphate and serves an important role in nucleotide synthesis. M*φ* utilize NADPH produced by PPP as electron carrier for ROS formation by NADPH oxidase (46) and as a co-substrate with l-arginine for the synthesis of l-citrulline and NO by inducible nitric oxide synthase (iNOS) (47). l-citrulline can also be involved in l-arginine biosynthesis to feed the cyclic and continuous NO generation in proinflammatory M*φ* (48). With regard to mitochondria, a decline in bioavailability of NAD^+^ due to depletion of tryptophan (co-substrate for NAD^+^ biosynthesis) or decreased influx from malate-aspartate and the glycerol-3-phosphate shuttles can disturb the NAD^+^/NADH ratio. Alternatively, an accumulation of NADH in the mitochondria due to decreased OXPHOS requirement results in disturbances of the NAD^+^/NADH ratio. Such flux in NAD^+^/NADH can disturb mitochondrial membrane potential and ETC complexes and consequently causes increased leakage of electrons to O_2_ favoring O_2_•^−^ production in M*φ* (49–52). Besides ETC, *α*-ketoglutarate dehydrogenase and pyruvate dehydrogenase complexes are also recognized as site of mtROS production (53, 54).

### Oxidative Metabolism in Immunomodulatory Macrophages

Immunoregulatory (M2 type) M*φ* rely on TCA cycle and OXPHOS sourced from FA uptake and lipids/FA oxidation to meet the energy demand (55). M2 M*φ* depend on adenosine monophosphate-activated protein kinase (AMPK) and peroxisome proliferator activating receptors (PPARs) mediated activation of STAT6 transcription complex to promote the expression of genes for OXPHOS and FA oxidation pathways (56). Treatment with chemical inhibitors of OXPHOS pathway or of ATP synthase downregulated the M2-specific expression of genes (*e.g. Arg1, Mrc1*), surface markers (CD206), and arginase 1 activity in IL-4-stimulated M*φ* (35). Conversely, LPS + IFN-*γ*-stimulated M1 M*φ* lacked the competence to restore mitochondrial respiration and M2-specific receptor expression after IL-4 treatment (35). Collectively, current literature suggests that M2-like M*φ* have an intact TCA cycle, FA oxidation, and aerobic glycolysis, while M1-like M*φ* tend to depend on breakdown intermediates of glycolysis, PPP, and TCA cycle.

### Metabolic Regulation of Macrophages in Infection

Very few studies have addressed the metabolic regulation of M*φ* polarization in experimental models of health and disease, though metabolic perturbations elicited in M*φ* by various infectious agents have been reported. For example, *Mycobacterium tuberculosis* invasion of M*φ* was accompanied by increase in expression of glucose transporters and glucose uptake (57). Further, *M. tuberculosis* augmented the aerobic glycolysis that was associated with delayed apoptotic response of M*φ* and increased replication and survival of the bacteria in M*φ* (57). In contrast, HIV dampened the glucose uptake, glycolysis, and PPP intermediates to inhibit proinflammatory M*φ* activation (58). Likewise, *Francisella tularensis* suppressed the lactate and HIF-1*α* stabilization and aerobic glycolysis in M*φ* to ensure early replication of the bacteria (59). *Salmonella* infection activated M2 M*φ* polarization despite the PPAR-*δ* mediated increase in glucose availability and favored the bacterial replication (60, 61). Pathogenic protozoans survive in M*φ* and use M*φ* as a vehicle for dissemination. For example, *Leishmania* sps. and *Toxoplasma gondii* utilized arginine metabolism for the synthesis of polyamines for their growth and replication in M*φ* (62, 63). *Leishmania* also enhanced the AMPK/Sirtuin 1 (SIRT1) activity that supports mitochondrial metabolism (64) and levels of M2-associated intracellular metabolites (65), while glucose consumption was decreased in infected M*φ* (65). *Trypanosoma cruzi* infection enhanced the expression of peroxisome proliferation activated receptor *α* (PPAR-*α*) in M*φ* (66), and PPAR isoforms have been implicated in the gene transcription roles in M2 M*φ* by several research groups (67). The cruciality of the PPP for providing substrates for ROS and NO production to control *T. cruzi* in M*φ* was also noted (66). Others have indicated that PPP production of NADPH is utilized by *T. cruzi* to activate its antioxidant enzymes (*e.g.* trypanothione reductase) as defense against ROS/NO produced in M*φ* (68). Together, these studies indicate that infectious agents employ various strategies to hijack the M*φ* metabolism machinery for their own survival and replication; and microbe-specific studies identifying the steps in metabolic pathway(s) and/or metabolites that can be targeted to enhance the pathogen clearance by M*φ* are required.

## Other Mitochondrial Pathways Inter-Linked With MACROPHAGE Response to Pathogens

Besides metabolic control, the mitochondria play several other key regulatory roles in shaping the M*φ* response and reviewed in brief here.

### Apoptosis

In conditions of stress, a feedback cycle of mitochondrial permeability transition (MPT), disturbance of electrochemical proton gradient across the respiratory chain complexes and mtROS production occurs. The mtROS and ROS-induced oxidants result in oxidation of glutathione and thiol (*e.g.* N-acetyl cysteine) pools that inhibit apoptosis (69) or through oxidation at mitochondrial membranes that generate MPT lead to cytochrome c (cyt c) release. Cytosolic cyt c interacts with apoptotic protease activating factor 1 (APAF-1) initiating recruitment and activation of caspase-9 and other effector caspases (caspase-3, -6, -7) leading to cell apoptosis (70, 71). Mitochondrial apoptosis-inducing factor and endonuclease G have also been identified as key signaling molecules of apoptosis (72) A wide range of pathogens, pathogen-shed vesicles, and bacterial toxins have been associated with mitochondrial disturbances, cyt c release and activation of apoptosis in M*φ*. For example, *Shigella flexneri* invasion induced mitochondrial MPT and signaled cell death in M*φ* (73). *M. tuberculosis* was shown to induce Bax translocation to the mitochondria, MPT, and cytosolic cyt c release in infected M*φ* (73, 74). *Bacillus anthracis* lethal toxin and *Staphylococcus aureus* secreted pore-forming toxins signaled activation of NLRP1B (75, 76) and NLRP3 (77, 78) inflammasomes, respectively, which led to caspase-1 and IL-1β activation and M*φ* pyroptosis (highly inflammatory form of programmed cell death), though mitochondrial involvement was not reported.

### Mitochondrial Fusion and Fission

Mitochondrial dynamics (*i.e.*, repetitive cycles of fusion and fission) and mitophagy maintain mitochondrial homeostasis (79). For fusion, transmembrane dynamin related GTPase proteins named mitofusins (MFN1/MFN2) form dimers across the interface and tether mitochondria together. Mitofusins utilize GTP hydrolysis and redox signaling to induce conformation changes and enforce mitochondrial outer membrane fusion. Conversely, ubiquitination targets mitofusins for degradation that involves PTEN-induced kinase (PINK)1, Parkin, E3 ligase Huwe1, and Bcl-2 family members. Another dynamin related GTPase protein, optic atrophy protein 1 (OPA1) interacts with cardiolipin localized in cristae and intermembrane space to coordinate fusion of inner mitochondrial membranes. Mitochondrial fission involves dynamin related protein 1 (DRP1) and other adapter proteins that form a ring across the outer mitochondrial membrane. DRP1 is regulated by a variety of post-translational modifications, and organization of DRP1 ring offers conformational activation of GTPase activity leading to spiral constriction and separation of damaged and healthy mitochondrial portions (80). Fission 1 protein, regulated by ubiquitination by mitochondrial ubiquitin ligase, is involved in DRP1 binding, and it facilitates fragmentation of damaged portion of the mitochondria. Several other proteins, *e.g.*, mitochondrial fission factor (MFF), TNFR-associated protein 1 (TRAP1), and mitochondrial dynamics proteins (MID49, MId51) are also involved in the fission process, though their role is not fully explored (81).

Studies on the role of mitochondrial fission and fusion in pathogen control have yielded important results. An increase in mitochondrial fission and mtROS production that activated the NF-*κ*B mediated expression of proinflammatory mediators was observed in LPS-stimulated microglial M*φ (* 82). *Streptococcus pneumoniae* infection triggered mitochondrial fission allowing energy switch and enhanced mtROS production in M*φ* (83). *Helicobacter pylori *vacuolating toxin induced DRP1 activation, mitochondrial fragmentation, Bax translocation to mitochondria, and cyt c release, and it was suggested that *H. pylori* targets mitochondrial fragmentation and apoptosis to prevent proinflammatory M*φ* response (84). Likewise, *Chlamydia trachomatis* maintained mitochondrial integrity and predominance of OXPHOS pathway through a miR-30c-5p-dependent inhibition of Drp1-mediated mitochondrial fission (85). *Vibrio cholerae* targeted mitochondrial Rho Miro GTPases to modulate mitochondrial dynamics and interfere with innate immunity (86). Overexpression of MFN2 increased the *M. tuberculosis* growth in M*φ* (87). However, a recent study showed that LPS-induced MFN2 was required for the adaptation of mitochondrial respiration to stress conditions and increase in ROS production and phagocytosis activity in M*φ* (88). M*φ* treated with MFN2 inhibitor or knocked down in MFN2 encoding gene exhibited a significant decline in phagocytosis of apoptotic bodies, *Aeromonas hydrophila* and *E. coli* (88). Studies in mice with myeloid-specific MFN1 and MFN2 knockdown revealed that MFN2 (not MFN1) deficiency impaired the production of proinflammatory cytokines and nitric oxide. MFN2 deficiency was also associated with dysfunctional autophagy, apoptosis, and phagocytosis, and MFN2 depleted mice failed to control *Listeria* and *M. tuberculosis* infections (88). These observations support the notion that mitochondrial dynamics is closely linked to the phagocytic capacity and immune function of M*φ* during infection.

### Mitochondrial Biogenesis

Mitochondrial biogenesis refers to the process by which cells increase mitochondrial mass (89). Peroxisome proliferator-activated receptor gamma coactivator-1*α* (PGC1*α*) plays an important role in the activation of nuclear respiratory factors (NRF-1, NRF-2) and nuclear factor erythroid 2-related factor 2 (Nfe2l2) transcription factors that regulate the expression of nuclear DNA and mtDNA encoded genes for mitochondrial biogenesis (90, 91). Recent discoveries indicate that mitochondrial biogenesis is intricately engaged with inflammatory/immunomodulatory host response. For example, it is suggested that early-phase inflammatory mediator proteins interact with pathogen recognition receptors to activate NF-*κ*B-, MAPK-, or protein kinase B/Akt-dependent pathways, resulting in increased expression and activity of cofactors and transcription factors involved in mitochondrial biogenesis [reviewed in (92)]. Nitric oxide and ROS generated by M*φ* can stimulate PGC-1*α*/Nfe2l2 or redox-sensitive hemoxygenase systems, causing simultaneous induction of mitochondrial biogenesis and antioxidant gene expression and modulate inflammation (93, 94). Sirtuin 1 (SIRT1), a highly conserved member of the Sir2 histone deacetylases family, is also implicated in deacetylation of PGC1*α* at multiple lysine sites, consequently increasing PGC1*α* activity (95). Specifically, *Staphylococcus aureus* is shown to upregulate mitochondrial biogenesis through the upregulation of the PGC family (96, 97). The induction of mitochondrial biogenesis was dependent on TLR2 and TLR4, signifying that the innate immune function feeds into the regulation of mitochondrial health during bacterial infection (97). Plataki et al. (98) noted decreased ATP production, dysregulated mitochondrial gene expression, and decreased numbers of healthy mitochondria in aged adult M*φ* and lungs in response to *S. pneumoniae* infection and showed that treatment with an anti-fibrotic drug improved the mitochondrial mass and health in aged M*φ* and decreased the pulmonary edema in aged mouse lung during infection. Human cytomegalovirus** **also induced mitochondrial biogenesis accompanied with increased respiration, both of which were required for the viral replication (99). Summarizing, the complex network of pro- and anti-inflammatory pathways impact and, are impacted by, mitochondria. While some infectious agents exploit mitochondrial biogenesis to establish infection in the host, maintenance of mitochondrial biogenesis, health, and function, and cellular redox status is vital to host survival in unregulated acute inflammatory states such as sepsis.

### Mitophagy

Mitophagy is a process used for the removal of fragmented mitochondria, and detailed mechanism of mitophagy is discussed in a recent review (79). Briefly, mitophagy is triggered by outer membrane receptor proteins (*e.g.*, NIX/BNIP3L, BNIP3, and FUNDC) that have a classic motif to bind directly to microtubule associated light chain 3 (LC3) and initiate mitophagy (100). Mitophagy is also initiated by the PTEN-induced kinase 1 (PINK1)/Parkin. Loss of membrane potential in damaged mitochondria supports the accumulation of unprocessed PINK1 on the outer membrane surface where it recruits cytosolic Parkin to promote ubiquitin dependent mitophagy through interaction with LC3 protein (101).

In context to cell damage caused by invading and intracellular microbes, autophagy/mitophagy act as important defense systems to establish homeostasis in stressful cellular environment. Indeed, inhibition of mitophagy by 3-methyladenine resulted in M*φ* polarization towards M1 phenotype (102), while rapamycin mediated induction of autophagy suppressed the mtROS and NLRP3 inflammasome activation and favored M*φ* polarization towards M2 phenotype (102, 103). Further, PINK1 depletion enhanced the LPS/IFN-*γ* stimulated proinflammatory phenotype in microglial cells (104), and adoptive transfer of *Pink1*-deficient bone marrow promoted M*φ* proinflammatory activation, which favored pathogen clearance and increased survival in a murine model of polymicrobial infection (8). However, PINK1 was required to trigger the RIG-I-mediated innate immune responses in M*φ* infected with respiratory syncytial and herpes simplex viruses (105), and Parkin-deficiency increased the susceptibility of mice to intracellular pathogenic bacteria (*e.g.*, *M. tuberculosis, S. typhimurium, L. monocytogenes*, and *Pseudomonas aeruginosa (* 106, 107 *).* Likewise, autophagy activation by isoniazid and bedaquiline treatment impaired the mycobacterial phagosome escape in M*φ*, suggesting that drugs favoring autophagy would enhance bacterial clearance (108). These studies highlight that mitophagy has important and diverse roles in modulating the innate immunity against pathogens.

## DNA Sensing by cGAS–Sting in Macrophage Activation

The cytosolic presence of mtDNA in M*φ* can occur due to uptake of fragmented/damaged mitochondria released in the peripheral system by other cells or due to leakage of mtDNA from their own mitochondria. The mtDNA contains a significant number of unmethylated CpG DNA repeats, similar to those present in prokaryotic bacterial genome (109) and is recognized as a cellular DAMP. The role of mtDNA as a key inducer of inflammatory cytokines (IL-1β and IL-18) in M*φ* through recognition by TLR9 and activation of NLRP3/ASC inflammasome has been recognized in previous studies (110, 111). The cytoplasmic mtDNA co-localizes with NLRP3 to induce IL-1β secretion and oxidized mtDNA is a potent inducer of IL-1β production in M*φ (* 112 *).* Further, NLRP3 activators trigger mtROS production, oxidized mtDNA release into cytosol and also mtDNA synthesis (without increase in mitochondrial mass) to fuel the proinflammatory activation in M*φ* (113, 114). Detailed information on mtDNA signaling of inflammasomes can be found in recent reviews (115–117).

The pivotal role of double-stranded DNA sensor cGAS and STING in shaping the immune-surveillance by M*φ* has been recognized recently (118). Briefly, STING was identified in 2008 (119) and cyclic guanosine monophosphate-adenosine monophosphate (cGAMP) was recognized as a novel secondary messenger serving as ligand of STING in 2013 (120). Subsequently, cGAS was noted to have cytosolic DNA-sensing ability, and the cGAS–STING pathway was identified to be indispensable for anti-viral host immunity (121). Later on, mtDNA and oxidized DNA were identified as key molecules to be recognized by cGAS–STING for the initiation of anti-viral type I interferon immunity (122). These authors showed that mtDNA stress elicited by a mitochondrial transcription factor A deficiency promoted mtDNA escape into cytosol where it engaged cGAS and signaled STING–IRF3-dependent increase in the expression of interferon stimulated genes (ISGs) in herpes viruses infection model (122). The findings that inhibition of DNA repair enzyme 8-oxoguanine DNA glycosylase 1, which removes 8-oxo-7,8-dihydroguanine lesions caused by ROS, enhanced the mtDNA-cGAS–STING–IRF3–IFN-*β* axis in favor of M*φ* control of *P. aeruginosa (* 123) showed that oxidized DNA is a more potent activator of cGAS–STING pathway. Indeed, impaired activity of DNA damage repair response mediators, such as ataxia telangiectasia mutated (ATM)-RAD3, poly ADP-ribose polymerase (PARP), and breast cancer1/2 (BRCA1/2) is associated with persisting double-stranded DNA breaks, accumulation of cytosolic DNA and activation of cGAS–STING pathway (124–126). The bone marrow derived dendritic cells and M*φ* from cGAS^−/−^, STING^−/−^, or IRF-3^−/−^ mice were deficient in anti-adenoviral responses (127, 128); however, cGAS^−/−^ and STING^−/−^ mice were not defective in the induction of adaptive immunity and achieved adenoviral clearance at a similar rate as was noted in WT mice (128).

Mechanistically, it was shown that activated STING binds to and be phosphorylated by TANK binding kinase 1 (TBK1) dimer or I*κ*B kinase (IKK) (129, 130) and it was suggested that STING–TBK1/IKK signalosome produces a scaffold to phosphorylate IRF3 (activate-form) or inhibitor of NF-*κ*B*α* (targeted for degradation) and consequently, signal IRF3-dependent type 1 ISGs and NF-*κ*B activation, respectively. TBK1 is also suggested to control STING stimulation. The p62/SQSTM1, which is phosphorylated by TBK1, directs ubiquitinated STING to autophagosome for degradation (131). Others indicated that caspase-9 and caspase-3 can cleave cGAS and IRF3 to restrain deleterious inflammation.

The cGAS–STING has also been reported to interact with the autophagy machinery. The STING–TBK1 activation and ISG expression induced ER stress and mechanistic target of rapamycin complex 1 (mTORC1) dysfunction (132, 133). Both ER stress and mTORC1 disturbances can signal Unc-51-like autophagy activating kinase (ULK1) and Beclin-1-class III phosphatidylinositol 3-kinase (PI3KC3) complexes, which together promote initiation of the classical autophagy (132, 133). cGAS was also indicated to directly interact with beclin-1-PI3KC3 complex to trigger autophagy (134). Further, cGAS–STING-mediated inflammation was mitigated by PINK1 and Parkin, which promote the clearance of damaged mitochondria (135). Some studies have also implicated the crosstalk between cGAS–STING and autophagy in pathogen clearance. For example, STING recognition of extracellular mycobacterial DNA enhanced the bacilli delivery to autophagosomes, and mice with monocytes incapable of delivering bacilli to the autophagy pathway were extremely susceptible to infection (136). Direct interaction between cGAS and beclin-1 not only halted the innate immune response against herpes simplex virus-1 infection but also enhanced the autophagy-mediated degradation of cytosolic pathogen DNA to prevent excessive cGAS activation and persistent immune stimulation (134). These findings suggest that autophagy/mitophagy provide a negative feedback control on over-activation of the cGAS–STING-mediated inflammation.

## Non-Coding RNA Regulation of Mitochondrial Health and Its Potential to Influence Innate Immunity

A significant portion of the eukaryotes’ genome encodes for non-coding RNAs (ncRNAs) of different sizes. The ncRNAs regulate diverse biological processes, and their significance in regulation of mitochondrial metabolic function has been recognized recently. In this section, we briefly discuss the influence of ncRNAs in shaping the mitochondrial health and interpolate its potential effects in shaping the host innate immunity.

Short, non-coding micro(mi)RNAs (~22 bp) regulate the gene expression by binding to the promoters, 5′ and 3′ untranslated regions (UTRs), or open reading frame (ORF) of the targeted mRNAs. For example, miR-23a/23b miRNAs participated in the regulation of mitochondrial TCA cycle *via* activation of glutaminase enzyme (137), and miR-30 family members inhibited mitochondrial fission and apoptosis through suppressing the *p53* and *Drp1* expression (138). These miRNAs, if present in M*φ*, would potentially favor M2 phenotype. Conversely, several miRNAs suppress mitochondrial homeostasis and have a potential to favor M1 M*φ* phenotype. Examples include miR-696 that inhibited FA oxidation and mitochondrial biogenesis by PGC-1*α* (139), miR-210 (upregulated in hypoxic conditions) that blocked mitochondrial respiration by inhibiting *Isc* gene homologs encoding for the iron sulfur clusters (140), and miR-195 that directly inhibits SIRT3 implicated in regulating mitochondrial metabolism (141). The miR-762 was recently shown to translocate to the mitochondria and inhibit translation of ND2 subunit of mitochondrial complex I, which enhanced mtROS generation (142).

Non-coding circular RNAs (circRNAs) form a covalent bond between their 5′ and 3′ ends and exhibit significant resistance to exonucleases. A mitochondrial fission and apoptosis-related circRNA (MFACR) was recently shown to enhance mitochondrial fission and apoptosis in cardiomyocytes, and MFACR knockdown attenuated mitochondrial fission and myocardial infarction in mice (143). Steatohepatitis-associated circRNA ATP5B Regulator (SCAR) is located in the mitochondria, where it binds to ATP5B and suppresses mtROS generation (144). Authors also showed that mitochondria-specific delivery of circRNA SCAR alleviated meta-inflammation *in vivo* (144). Mitochondrial genome-derived mc-COX2 circRNA was present in plasma of chronic lymphocytic leukemia (CLL); a decline in mc-COX2 affected mitochondrial functions and induced cell apoptosis and its upregulation was positively associated with worsening survival of CLL patients (145). How mitochondria targeted circRNAs influence inflammatory *vs*. immunomodulatory role of M*φ* or other innate immune cells is not studied.

Long non-coding RNAs (lncRNAs; >200 nucleotides) bear many signatures of mRNAs, *e.g.*, 5′ capping and polyadenylation and can impact gene expression by interacting with DNA, RNA, or proteins (146). LncRNAs play a role in mitochondrial homeostasis by directly influencing the expression and/or stability of the mRNAs encoding for mitochondrial proteins or by indirectly affecting the miRNAs that may govern the half-life of mRNAs responsible for mitochondrial health. A few lncRNAs encoded by mtDNA include lncND5, lncND6, and lncCytb (147), though their role in mitochondrial function is not known. Recently, lncRNA Cerox1 (cytoplasmic endogenous regulator of oxidative phosphorylation 1) was identified to enhance the mitochondrial complex I activity by blocking regulatory effects of miR-488-3p on mRNAs for complex I subunits (148). Tug1 (taurine-upregulated gene 1) lncRNA regulates PGC-1*α* activity, and its overexpression enhanced the mitochondrial bioenergetics in the podocyte of diabetic nephropathy (149). Several lncRNAs have been identified to regulate apoptosis through their influence on mitochondrial health. For example, inhibition of AK055347 lncRNA was detrimental to cells’ viability that was accompanied by downregulation of ETC, cyt P450, and ATP synthase (150). Cardiac apoptosis-related lncRNA (CARL) blocked mitochondrial fission and apoptosis by impairing miR-539-dependent PHB2 downregulation (151), while FAL1 lncRNA enhanced DRP1-mediated mitochondrial fission and apoptosis (152). GAS5 and SAMMSON lncRNAs are also shown to regulate apoptosis *via* diverse mechanisms.

Overall, it is becoming increasingly clear that ncRNAs, originated either from nuclear or mitochondrial genome, are involved in regulating the mitochondrial homeostasis and will have a role in shaping the innate immunity.

## Summary

In this review, we have summarized the current literature on various roles of mitochondria in influencing the M*φ* response. It is well recognized that the mitochondria can satisfy cell energy requirements by maintaining their dynamicity, and alterations in mitochondrial structure and dynamics can modulate M*φ* immune response. The various mechanisms applied by pathogens to perturb and reprogram M*φ* metabolic health in favor of their survival in the host are also appreciated in the current literature. We envision that novel therapeutic strategies targeting mitochondrial dynamics will be useful in controlling the pathogenic effects of the overly active immune system while achieving the beneficial effects against the intracellular pathogens.

## Author Contributions

SC wrote first draft, IC wrote the section on ncRNAs, and SC and NG contributed to editing and writing of the final version. All authors contributed to the article and approved the submitted version.

## Funding

This work was supported by a grant from the National Institute of Allergy and Infectious Diseases (R01AI136031) of the National Institutes of Health to NG. The funders had no role in the study design, data collection and analysis, decision to publish, or preparation of the manuscript.

## Conflict of Interest

The authors declare that the research was conducted in the absence of any commercial or financial relationships that could be construed as a potential conflict of interest.
